# Factors determining the quality of health services provided to COVID-19 patients from the perspective of healthcare providers: Based on the Donabedian model

**DOI:** 10.3389/fpubh.2022.967431

**Published:** 2022-11-28

**Authors:** Malihe Sadat Moayed, Robabe Khalili, Abbas Ebadi, Akram Parandeh

**Affiliations:** ^1^Trauma Research Center, Nursing Faculty, Baqiyatallah University Medical of Science, Tehran, Iran; ^2^Behavioral Sciences Research Center, Nursing Faculty, Life Style Institute, Baqiyatallah University of Medical Sciences, Tehran, Iran; ^3^Department of Community Health, Medicine, Quran and Hadith Research Center, Nursing Faculty, Baqiyatallah University of Medical Sciences, Tehran, Iran

**Keywords:** pandemics, quality of health care, health personnel, delivery of health care, Donabedian model, COVID-19

## Abstract

**Objectives:**

The present study aims to explain factors determining the quality of health services provided to COVID-19 patients from the perspective of healthcare providers based on the Donabedian model.

**Method:**

This qualitative study was conducted at a referral hospital on COVID-19 patients in Tehran, in 2020. The data were collected through individual and semi-structured interviews from 20 participants using the purposive sampling method. Besides, data analysis was conducted simultaneously using the directed content analysis method.

**Results:**

Data analysis results produced 850 primary codes in three predetermined categories of the Donabedian model, including the structure (organizational readiness and continuous training), the process (effective management and leadership, safe care, and comprehensive care measures) and outcomes (professional excellence, quantitative and qualitative improvements in hospital services, and acceptability of healthcare professionals).

**Conclusion:**

The results of this study can help managers better understand how a public health crisis affects the structure of organizations providing care and treatment, quality of treatment processes in the organization, and the consequences. In addition, this study can be used as a model for optimizing the structures and processes to improve outcomes.

## Introduction

The prevalence of COVID-19 in the world, apart from creating a health crisis in healthcare systems, has changed economic, political, and social dimensions of society, being growing in some countries (1). In addition, the continuation of this trend in the health system has been overshadowing the quality of care services provided to patients (2). Providing quality services is a priority in the healthcare system. In most countries, the rating and accreditation of hospitals depend on the quality of care (3). In addition, providing systemic quality services can result in reduced organizational costs, increased productivity, enhanced employee satisfaction with services, and elevated client satisfaction (4).

The number of patients referring to medical centers during pandemics increases significantly, so the low quality of health services affects an organization's clients directly and indirectly (5, 6). Thus, giving due attention to providing high quality services and satisfying clients are more necessary than maintaining community health and creating loyal clients in the future (7). According to a study, even before the COVID-19 pandemic, healthcare organizations faced challenges in maintaining patient safety and providing quality care, especially during previous infectious crises. Therefore, they require careful and continuous evaluations and reviews to deal with the epidemic and to provide safe high quality care concurrently (2).

A previous studies in Iran showed that in the field of COVID-19 crisis management in the healthcare system, the issues of contact tracing and control of the chain of disease transmission are still neglected (8). In addition, high workloads among healthcare workers, inefficient management of equipment, and mental health issues are among the main challenges of the Iranian healthcare system (9). To evaluate and respond to such challenges, various models and criteria, such as “Donabedian”, “SERVQUAL”, “HEALTHQUAL”, “PubHosQual”, and “HospitalQual” have been introduced to measure the quality of health services in different areas (10). However, most healthcare quality models have been designed for normal situations, which may not be appropriate during all crises, such as the COVID-19 pandemic (11).

### The Donabedian model and its uses

From among the models available for evaluating the quality of care, the Donabedian model was selected for its simplicity, flexibility, and appropriateness in critical situations (12). The use of this model, both structurally and through expanding precautionary measures and personal protective equipment, can be used as a guide to improve the structure and process in initial responses to the COVID-19 crisis to achieve high quality clinical results (13). This model was introduced in 1966 as a framework for assessing the quality of health services. In addition to the developer of the care model, Donabedian is known as the pioneer in the study of the quality of care services (14).

According to Donabedian, both technical and interpersonal qualities of healthcare services are essential for improving quality of healthcare services. Technical care refers to the manner and dimensions of patient care, and interpersonal relationship care deals with communication with the patient about their care (12). It is generally accepted that quality of health care services should be measured according to explicit criteria reflecting the values of a particular society. In addition, it must be performed using the perspective of the main stakeholders, such as users, healthcare providers, service providers, politicians, and health managers (15).

The Donabedian model is a three-part model used to create a systematic framework for improving the quality of healthcare services. Donabedian maintained that measuring the quality of services would be ineffective as long as there were no reliable and valid tools. The Donabedian framework indicates that patient outcomes are affected by clinical care delivery structures and processes, both of which directly impacting COVID-19 pandemic outcomes. Thus, he presented his model in the three parts of the structure, process, and outcomes. In this model, the structure functions as spatial features in which healthcare takes place, being like the architecture and availability of equipment. On the other hand, the process includes providing care to patients as well as the workflow. In addition, the outcomes describe effects of healthcare on the population and returning patients to their initial position or surviving them (16).

The use of this model, both structurally and by expanding precautionary measures and personal protective equipment, can be used as a guide for improving the structure and process of initial responses to the COVID-19 crisis to obtain high quality clinical results (13). The results of a case report on the first wave of the COVID-19 pandemic in New York in a hospital emergency department showed that this model could be organized so as to achieve desired safety outcomes in patients and staff (13). Another study described specific structures, processes, and outcomes as barriers or factors affecting the quality of care (17).

Although few studies have been conducted on the quality of services during the COVID-19 pandemic, it is assumed that the emergence of COVID-19 and differences in healthcare systems (based on the level of developments in each country) require us to conduct a qualitative study on patients' experiences and perceptions (18). Qualitative research, by exploring deep and valuable experiences of main stakeholders regarding healthcare services, mitigates the impact of intervening factors in the structure, process, or outcomes of services, thereby improving the quality of healthcare services (19). In fact, conducting a qualitative study using this model, based on process-oriented and action-oriented measures, can be effective in evaluating factors determining the quality of healthcare services provided to COVID-19 patients from the perspective of healthcare providers. There is no research in Iran on healthcare workers' experiences during the COVID-19 pandemic. Thus, the results of such studies can help optimize healthcare structures, nursing care processes, and patient outcomes during the current epidemic to prevent future public health emergencies. Therefore, given the spread of different variants of COVID-19 and challenges facing the healthcare system, the present study was conducted to explain factors determining the quality of health services provided to COVID-19 patients from the perspective of healthcare providers based on the Donabedian model.

## Method

The present qualitative study was conducted at a referral hospital for COVID-19 patients in Tehran, Iran, in 2020. This hospital had the highest number of COVID19 visits in statewide. Various treatment modalities were used, including antivirals, hemoperfusion, plasma exchange therapy, extracorporeal membrane oxygenation (ECMO), etc. This study was guided by the Quality Framework for Evaluation of Healthcare Delivery (20). The Quality Framework informed the interview guides, data analysis, and the research question, including the rationale for exploring structure- and process-based aspects of healthcare.

To this end, a total of 20 people, including 17 healthcare providers (specialists, nursing managers, nurses, assistant nurses, and nursing students) were selected using the purposive sampling method. During the participant selection process, maximum possible diversity was achieved in terms of the variables of age, gender, education level, clinical work experience, specialization, and organizational position. To complete triangulation in data sources, two recovered COVID-19 patients and a caregiver's family were included in this study.

The inclusion criteria were employment in COVID-19 patient care units, being experienced in providing direct COVID-19 care to patients based on the purpose of the study, being able to transfer information, and being willing to participate voluntarily in the study. Participants with reportedly physical or psychological health problems, or those reluctant to continue the interview were excluded from the study. None of the participants withdrew from the study after announcing their agreement on participation. To collect data from individuals, in-depth face-to-face and semi-structured interviews were conducted to discover their experiences in the 6-month period from May 21, 2020 to November 20, 2020. In fact, data collection and analysis were conducted concurrently. To conduct the study, the researcher was present in the research environment. Besides, while formally introducing himself and explaining the purpose of the study to the participants, he made necessary agreements with them on receiving information and conducting face-to-face interviews.

The interview environment was chosen based on the participants' choice, in a completely quiet environment, such as the working room or the resting area of healthcare providers. The interview lasted 30–90 min based on the healthcare provider's tolerance, information, willingness, and agreement. During the semi-structured interview, the healthcare providers were asked questions about the concepts of the model. Accordingly, they were asked to give information about experiences of care methods, problems during providing care, effects of care on the treatment process, and their role. The interviews were conducted by two researchers, including the first author and the corresponding author, as well as a clinical nurse with a doctorate degree, sufficiently experienced in qualitative studies.

At the beginning of each interview, the interviewers introduced themselves and briefed the participants on the purpose of the study. In addition, oral consent and written consent forms were obtained from the participants. Next, the semi-structured interview process was followed and continued until data saturation with purposeful questions about main concepts of the Donabedian model, including the structure, process, and outcomes. To keep the interviews uniform, some guiding questions were prepared for all sessions. The guide included interview questions about healthcare providers' experiences, descriptions of the critical care work system structures, care processes, outcomes, and the impacts of the COVID-19 pandemic. In addition, the interview guide was revised by the research team and pilot tested with three healthcare workers who met the inclusion criteria, yet they did not participate in the study. Each interview began with one of the following general items of “*Please describe your experience of caring for COVID-19 patients.”*, “*Please describe a day you cared for a COVID-19 patient.”*, or “*What problems did/do you face in the management of COVID-19?”* The interview guide is reported in Supplementary File 1.

In the meantime, some exploratory questions, such as “What do you mean?” and “Would you please explain more?” were asked to discover the hidden aspects of the participants' experiences and to enrich the interview findings. To achieve data saturation, three new interviews were conducted with the healthcare providers, which ended with 20 participants.

In addition, directed or pattern-based content analysis was used to analyze the data. This method, introduced by Hsieh and Shannon (21), is suitable for qualitative analysis of texts, which uses a theory or a model. In directed content analysis, initial coding begins with a theory or similar research findings, with its purpose being to validate or develop a previous conceptual or theoretical framework or model. In the data analysis process, based on the existing theory or model, additional codes may be developed, with the original design revised or corrected (21).

In this study, the known key concepts of the Donabedian model were considered the main categories. Next, operational definitions of the concepts were extracted for each category, according to the Donabedian model. Besides, all interviews were recorded after obtaining consent from the participants. In addition, data analysis was started by reading the full text of the interview several times to gain an overview of the state of the care provided during the COVID-19 pandemic. In fact, the codes were placed in the pre-identified categories of the model based on conceptual similarities, with the concepts of the subcategories produced based on the range and logical relationship of the data in the categories. Data not fitting the main categories of the model were replaced in a new category. Upon repeatedly reviewing the data, new categories were named based on the content of the codes, with their subcategories identified by further analysis. Next, initial operational definitions were provided.

Lincoln and Guba's (1986) criteria were used to improve the trustworthiness and rigor of the data (22). Next, a sample of the texts was reviewed and approved by the participants (member checking), including two healthcare providers and a clinical nurse experienced in caring for COVID-19 patients (peer checking). In addition, the data analysis process was reviewed by two researchers with qualitative work experience and prolonged engagement in research credibility. Besides, data transferability was assessed through selecting the participants with maximum diversity and accurately describing the details of the study for the purpose of transparency. Furthermore, dependability was assessed by continuous comparative data analysis and data triangulation through interviews with major and minor participants. Finally, the documents were recorded over time for confirmability. In the coding process and in the course of categorizing methodological coherence, external checking and peer briefing were performed.

Ethical considerations were taken into account upon obtaining ethics code BMSU.REC.1399.026 from the Research Ethics Committee of the university. The participants were briefed on the objectives of the study to obtain their informed consent. In addition, the people's names were written as codes in the data report for confidentiality purposes. Besides, the place and time of the interview were determined upon agreement with the participants. The participants were allowed to quit the study whenever they wished.

## Results

Out of the 20 participants in the present study, 6 and 14 were female and male, respectively. The mean age and work experience of 17 participants were 38.29 ± 8.85 and 11.29 ± 9.02 years, respectively. Besides, their education level varied from a high school diploma to a specialty degree in medicine. All of the participants were active during the COVID-19 pandemic. In addition, two patients and a patient companion, including two females and one male with the mean age of 46.66 ± 55.20, were among the other participants of the study (Table 1 shows the participants' demographic characteristics).

**Table 1:** Demographic characteristics of the participants.

**Number of the participant**	**Sex**	**Age**	**Education**	**Job**
1	Male	47	PhD	Nurse/Faculty member
2	Male	38	PhD student	Nurse
3	Male	47	PhD	Nurse/Faculty member
4	Male	36	MSc	Nurse/Faculty member
5	Male	55	MSc	Nurse/Faculty member
6	Male	35	MSc	Nurse
7	Male	30	Associate's	Nurse
8	Female	38	Bachelor's	Nurse
9	Female	28	Bachelor's	Nurse
10	Male	27	MSc student	Nurse
11	Male	40	Bachelor's	Nurse
12	Male	32	Associate's	Nurse
13	Male	50	Emergency medicine specialist	Physician/Faculty member
14	Male	39	Bachelor's	Nurse
15	Male	42	Emergency medicine specialist	Physician/Faculty member
16	Female	22	Bachelor's	Nurse
17	Female	45	Associate's	Nurse
18	Male	57	Diploma	Companion
19	Female	60	Diploma	Patient
20	Female	23	Bachelor's	Patient

The findings revealed 850 primary codes that were classified in 8 categories and 22 subcategories. Given the unobtrusiveness of the directed approach in data analysis, all identified categories were placed in the class matrix derived from the Donabedian model. In the end, all resulting codes and categories were placed under the three dimensions of structure, process, and outcomes, being the components of the Donabedian model (Table 2).

**Table 2:** Main categories, generic categories, and subcategories obtained from the direct content analysis.

**Dimension**	**Main category**	**Sub category**
Structure	Organizational readiness	Crisis management
		Appropriate infrastructures
		Benefiting from international experiences and assistance
	Continuous training	Training and maneuvering programs
		Trained personnel
Process	Effective management and leadership	Human resource planning
		Organization and coordination in the supply and distribution of human resources
		Decision-making skills during crises
		Professional communication during crises
		The need for a united command
		Employing voluntary forces
	Safe care	Inadequate and unsuitable protection conditions
		Inadequate environmental health conditions
	Comprehensive care measures	Focusing on emotional needs
		Care as a professional responsibility
Outcome	Professional excellence	Competency promotion
		Self-confidence
		Professional promotion
	Quantitative and qualitative improvements in hospital services	Structural development of hospitals
		Promotion of specialized medical and educational services
		Equipping hospitals with medical supplies
	Acceptability of healthcare professionals	Patient trust
		Patient satisfaction

Accordingly, “Organizational readiness” and “continuous training” constituted the dimension of structure. In addition, “effective management and leadership”, “safe care”, and “Comprehensive care measures” made up the dimension of process. Besides, “professional excellence”, “quantitative and qualitative improvements in hospital services”, and “acceptability of healthcare professionals” formed the dimension of outcome (Supplementary File 2).

Each main category is explained below using the participants' direct quotations.

### The first dimension: *Structure*

The dimension of structure included two categories of operational preparedness and continuous training with the following subcategories:

### Main category 1: Organizational readiness

Having an action plan or being prepared is a key indicator of crisis management and control. This category included the 3 subcategories of “*crisis management”, “appropriate infrastructure”*, and “*benefiting from international experiences and assistance”*.

#### Subcategory 1: Crisis management

Crisis management refers to making necessary organizational arrangements to prevent, address, and deal with a crisis or to minimize its destructive effects. One of the basic structures for effectively dealing with a crisis is to provide the context and to make prior preparations. Performing effective crisis management requires prevention, preparedness, response, and reconstruction strategies. Such preparations must be made for various aspects of management, personnel, organization, equipment, and physical space. Appropriate structures, crisis management plans, preparedness of medical departments for providing large-scale care, lack of a coherent plan for biological crises, as well as prevention and preparedness strategies for reducing COVID-19 crisis outcomes were among the codes repeated by the participants.

Concerning determination of structures appropriate for the crisis, participant 5 stated:

“*The structure has to be defined in advance, individuals must be organized, and responsibilities have to be determined. If there is an accident or a crisis, at this stage, the people will be in place and everything will be going well. However, during the COVID-19 crisis, we solved many problems quickly, but this will not always be the case”*.

Participant 10 talked about poor performance in the fields of crisis management and training crisis managers:

“*The important thing was that none of the hospitals, wards, doctors, and staff members was prepared, which worsened the situation. In terms of management, if we had a framework formulated, most of our patients would survive”*.

#### Subcategory 2: Appropriate infrastructures

To deal with crises, such as contagious diseases, the presence of buildings and adequate physical space is among the basic requirements of the ability to receive patients and provide quality services. The participants considered it necessary to have multiple therapeutic and modifiable treatment facilities to provide care in the large-scale wave of COVID-19 patients.

In this regard, participant 15 stated:

“*The NBC ward has helped us a lot and has always supported us in different challenges and crises we face in this country”*.

Participant 3, on the other hand, talked about the unsuitable design of the ward for providing services and maintaining health of the medical staff's health:

“*Here in the CCU, the station is placed at the center, and the beds are circular. Besides, 14 patients cough, who are all towards the center where the station is placed”*.

#### Subcategory 3: Benefiting from international experiences and assistance

Using other countries' experiences can be effective in fighting emerging diseases with unknown treatments. In this group of diseases, using a trial and error method may cost countless patients their lives.

Participant 15 said:

“*Using international experiences, like biological debates, is something new, which requires more research. However, in biological debates, I gathered information on challenges we had and used experiences from other parts of the world. Accordingly, we made necessary changes to fight this pandemic.”*

Participant 13 talked about the lack of information on applying protocols:

“*At the beginning, our information and protocols were incomplete for starting treatment, so we had to adopt protocols from other countries, like China”*.

### Main category 2: Continuous training

Providing continuous training to employees is one of the requirements of the success and promotion of medical services. Providing facilities and the context required for providing training and programs, as well as the presence of trained personnel are the factors effective in in the dimension of structure.

#### Subcategory 1: Trained personnel

Providing timely, correct, and appropriate services during crises requires scientific and practical knowledge of all health workers. Accordingly, using trained personnel in hospital wards can help improve the treatment process.

In view of this, participant 15 stated:

“*We expect the emergency acute care or the NBC to provide staff with higher capabilities and higher academic levels, being still a shortcoming. Hospitals and universities need to focus more on this issue”*.

#### Subcategory 2: Training and maneuvering programs

Running training courses and performing maneuvers on dealing with all kinds of crises enhance medical staff' skills and preparedness. Besides, such programs are effective in providing better services to patients and improving disease outcomes.

In this regard, participant 15 continued:

“*We have already performed several maneuvers. However, we have not experienced real public health crises. Public health debates are somehow new”*.

Accordingly, participant 10 stated:

“*Assessing needs, running a variety of training programs, and performing relevant maneuvers could help manage COVID-19 patients better”*.

### The second dimension: Process

This dimension includes the measures of effective management and leadership, safe care, and comprehensive care, which emerged from the data analysis. It is worth noting that consideration of other factors, such as organizational management and leadership as well as therapeutic processes, are vital in this category.

### Main category 1: Effective management and leadership

This category includes the subcategories of “human resource planning”, “organization and coordination in the supply and distribution of human resources”, “crisis decision-making skills (process)”, and “professional communication in crises”.

#### Subcategory 1: Human resource planning

Planning and determining correct arrangement of personnel in the medical sector were among the most important capabilities in the COVID-19 pandemic. Participant 8 referred to the planning method and the ignoring of requests made by nurses for choosing work shifts as the main sources of fatigue and dissatisfaction:

“*Ever since I joined the NBC, I have not had the right to choose a work shift. On the first day, the shift manager told me that they needed a 24-hour shift nurse, which created some difficulties at least for me”*.

Turning treatment wards into COVID-19 wards and the employment of personnel with different specialties and limited experience in the COVID-19 ward aggravated this issue even more than before.

#### Subcategory 2: Organization and coordination in the supply and distribution of human resources

Proper supply and distribution of human resources are major processes in providing services to patients, being among the most challenging processes in controlling the COVID-19 pandemic. In this regard, participant 10 said:

“*Personnel with no or little commitment to the system, like students, went on a sick leave. On the other hand, there was the problem of the large number of human resources. For instance, there were sometimes four to five health workers in a work shift with 10 patients, which were not needed at all”. This condition indicated inefficacy in the distribution and organization of nurses*.

#### Subcategory 3: Decision-making skills during crises

Decision-making management in critical situations is not possible for everyone. Accordingly, it is only possible when one has enough experience. Thus, under such conditions, there is a need for specialized people having decision-making skills.

On this, participant 5 said:

“*Most of the nursing managers were young and inexperienced, yet our previous managers were experienced in earthquake and war conditions, which was very helpful. Unfortunately, the managers did not have necessary crisis management and decision-making skills, being the reason that they did not perform well”*.

Based on the participants' experiences, mere youthfulness could not be enough for appointing managers; in fact, this could be associated with many problems under critical conditions. On the other hand, inability to guide COVID-19 patients was another challenge posed by the lack of decision-making skills in managers in COVID-19 wards. Therefore, COVID-19 crisis management requires people experienced in crises, having been challenged by the shortage of experienced managers to achieve this goal.

On this subject, participant 5 stated:

“*We had problems guiding COVID-19 patients. For instance, a patient suspected of COVID-19, who should have been admitted to the emergency respiratory ward could contaminate the emergency room; this required more attention from the shift manager”. Therefore, specialized managers are required to know how to triage and isolate patients*.

#### Subcategory 4: Professional communication during crises

Optimal inter-professional communication is a strength in providing quality services to patients. In addition, it is effective in the correct and timely diagnosis as well as the proper care provided by the treatment team. In this study, the presence of this skill resulted in numerous consequences.

On this point, participant 9 stated:

“*Teamwork increased the use of information in every field; in addition, interactions, like warmly welcoming the patients, led to providing better care”*.

#### Subcategory 5: The need for a united command

As a general rule, the presence of a single and united command for issuing all orders is effective during a crisis. Besides, it is helpful for everyone to know who or which team makes the final decision so as to listen to the command of a single leader to control the crisis.

“*Our major problem was the large number of bosses and leaders. In fact, there was no unity of command, with everyone issuing orders by themselves”, participant 6 said. This issue caused lack of coordination and loss of resources*.

#### Subcategory 6: Employing voluntary forces

Among the main responsibilities of an effective leader for controlling and managing a given crisis, one can refer to inviting and leading voluntary forces as complementary measures in providing services. However, this measure was not adopted in line with the framework of specific rules, yet it was formed spontaneously among voluntary groups.

In this respect, participant 17 said:

“*Voluntary groups, Basij forces, and Islamic theology students showed up, with some of whom having been very skillful. Accordingly, they assisted us very well, and their spiritual and religious support had a positive effect on the patients”*.

### Main category 2: Safe care

Providing care under unsafe conditions was among the problems in the first wave of the COVID-19 pandemic. This category included the two subcategories of “inadequate protection conditions” and “inadequate environmental health conditions”.

#### Subcategory 1: Inadequate protection conditions

Providing care without standard adequate PPE at the onset of the COVID-19 pandemic limited the medical staff.

Regarding PPE, participant 4 said:

“*In fact, we needed drinks, our mucus membranes would dry out, our body would become dehydrated, and we would sweat a lot. Most of my colleagues drank so infrequently that they would not need to go to the restroom; this in turn caused lethargy and made our mucus dry”*.

#### Subcategory 2: Inadequate environmental health conditions

According to the existing assumptions, having contact with contaminated surfaces leads to disease transmission. In addition, delayed arrival of hospital environmental health units to disinfect wards was one of the major problems mentioned by the participants.

Accordingly, participants 13 said:

“*The disinfection performed was unprincipled and inadequate. They did not observe hygiene procedures themselves, which made them susceptible to contract the disease. Unfortunately, the health unit started working very late.”*

### Main category 3: Comprehensive care measures

During a crisis, maintaining consistent care standards is one of the major treatment challenges in providing appropriate services to patients.

#### Subcategory 1: Focusing on emotional needs

Owing to the severity of anxiety and fear, due attention was not given to the patients' emotional and psychological needs in the first wave. As soon as the people became aware that someone was infected with COVID-19, all individuals around the patient stood away from them and minimized communication with them after hospitalization. Accordingly, visiting the patients was forbidden, with their needs neglected by health workers due to the heavy workload.

In this respect, participant 4 said:

“*Some doctors or nurses are afraid to go to a patient, or they keep their distance from the patient when approaching them. In fact, the patient would like to hear the doctor's answers to some of their questions”. Under such conditions, optimal communication was minimized between the patient and health workers*.

#### Subcategory 2: Care as a professional responsibility

The participants considered it their professional and human responsibility to provide care services to patients in all circumstances. This responsibility included providing the introduced treatments to COVID-19 patients as well.

Regarding this issue, participant 1 said:

“*We just did what we were supposed to do. We were assumed to do our responsibility, not to care for the results. Accordingly, we provided the care, which worked well.”*

### The third dimension: Outcomes

In this study, the outcome consisted of professional excellence, quantitative and qualitative improvements in hospital services, and patient satisfaction.

### Main category 1: Professional excellence

Although the occurrence of a crisis is the main cause of damage as well as loss of life and money, it can lead to the growth of organizations and individuals as well. In the data analysis, this category included the three subcategories of “*competency promotion”, “self-confidence”, and “professional status promotion.”*

#### Subcategory 1: Competency promotion

Coping with a crisis increases professional competence and capabilities. This promotion is due to the experience in providing care, the training provided, and the learning acquired. All these factors lead to the acquisition of unique skills and abilities.

Concerning this factor, participant 10 stated:

“*We were not used to knowing much about a ventilator, but in COVID-19 conditions, we have come to the conclusion that we need to learn more and improve our skills and abilities. Accordingly, the guys working here and those in many other wards have improved scientifically”*.

#### Subcategory 2: Self-confidence

When providing care, the healthcare providers came to the conclusion that they could play a vital and irreplaceable role in treating patients. In fact, this result might be achieved by losing one's life.

In this connection, participant 12 said:

“*Nurses believe in their own abilities. We, as nurses, can interpret chest x-rays, CT scans, and many other things better right now, while we did not have such skills and abilities in the past”*.

#### Subcategory 3: Professional promotion

The health team's altruistic services and unique role in caring for patients changed everyone's attitude, especially toward nurses.

On this issue, participant 10 stated:

“*The society's view has changed towards nurses and nursing. Everyone has reached the conclusion that a nurse can play a leading role in providing treatment, and that treatment is not merely provided by doctors”. This outcome was very desirable for the participating nurses*.

### Main category 2: Quantitative and qualitative improvements in hospital services

Other outcomes of the care under this category include “*structural development of the hospital”, “promotion of specialized hospital services”*, and “*equipping the hospital with medical supplies”*.

#### Subcategory 1: Structural development of the hospital

Increasing the number of specialized beds and adding treatment spaces were among the cases needed in the field of structural development of the hospital. One of the major structures designed and established was the provision of several large recovery areas and a significant number of beds for transferring COVID-19 patients to them.

In this regard, participant 10 stated:

“*Although the construction of recovery areas created some problems, it provided a great environment for patients to stay in when infected so that they would not infect any family members”*.

#### Subcategory 2: Promotion of specialized medical and educational services

Organizing professional respiratory teams and using up-to-date equipment were the other complementary moves added to the set of facilities provided to patients in the COVID-19 pandemic. In addition, using trained specialists as well as holding seminars and webinars were other measures adopted to improve scientific and practical skills in this respect.

In this regard, participant 17 stated:

“*We are setting up workshops to increase capabilities of our nurses and doctors; besides, we constantly provide training and check our facilities”*.

#### Subcategory 3: Equipping the hospital with medical supplies

Equipping the hospital with facilities, such as high-flow oxygen generators, various ventilators of different functions, and various protective equipment was another consequence of the COVID-19 pandemic.

On this issue, participant 14 said:

“*The reduction in the pressure and amount of the oxygen existing in the hospital caused us to set up large oxygen tanks or disposable clothing production workshops”*.

### Main category 3: Acceptability of healthcare professionals

Our data analysis results indicated “patient trust” and their “satisfaction” in the services provided by the hospital.

#### Subcategory 1: Patient trust

Since people considered medical staff as the individuals sincerely sacrificing their lives, being engaged in providing services in these circumstances, they accompanied them with confidence in following the treatment process.

“*I had not experienced such a situation in my entire life. Despite the huge crowd of the patients, they would attend the emergency room in complete peace and respect, without being upset or distressed”, participant 13 stated*.

#### Subcategory 2: Patient satisfaction

Our data analysis results indicated patient satisfaction and their trust in the services provided by the hospital. Since people considered medical staff as the individuals sincerely sacrificing their lives, being engaged in providing services in these circumstances, they accompanied them with confidence in following the treatment process.

“*I had not experienced such a situation in my entire life. Despite the huge crowd of the patients, they would attend the emergency room in complete peace and respect, without being upset or distressed”, participant 13 stated*.

In addition, patients and their families constantly accompanied the medical staff with their blessing and benediction, and prayed for their well-being, having been completely satisfied with their performance.

Considering this issue, participant 19 (a patient's companion) said:

“*May God bless them; they disconnected my patient from the device despite the severity of the disease, yet we are now heading home. Under such conditions, I did not really think that he would survive”*.

In contrast, some patients and their families were severely dissatisfied with the lack of recovery of their patients; accordingly, their expectations of the treatment were not met at this center.

In this regard, participant 18 (patient) said:

“*We thought they would provide COVID-19 treatment in this hospital. This is the reason why we just came here, yet we realized that they did not give the main COVID-19 treatment”*.

## Discussion

The present study aimed to identify the factors determining the quality of health services provided to COVID-19 patients from the perspective of healthcare providers and based on the Donabedian model. In this study, we found eight subcategories in the three dimensions of the structure, process, and outcomes.

In a study investigating the integration of e-health and healthcare based on the Donabedian model, the categories of the dimensions of this model were introduced. Accordingly, the internal environment, care recipients, and technology made up the dimension of structure; technical activity, interaction between healthcare professionals, and process management constituted the dimension of process; furthermore, the dimension of outcome included health status, satisfaction (experiences of patients and healthcare providers), and efficiency.

The results of the aforementioned study were consistent with those of the present one. The internal environment in the aforementioned study can be considered equivalent to organizational preparedness in our study. However, the literature used to address the urgency and COVID-19 threats influenced the way words were used in the present study. In the study by Tossaint-Schoenmakers, a patient was considered one of the major components of the structure of the Donabedian model, exerting a significant effect on other components of the structure (23). In our study, despite the effect of COVID-19 on all aspects of the Donabedian model, such a subcategory was not found in the data analysis structure.

Professional communication was another subcategory of our model for the dimension of process. In line with the present study, in other studies, professional communication was composed of one of the subcategories of the dimension of process (13, 23). Outcomes in the present study included promoting hospital services and patient satisfaction. Moreover, performance, health status, and patient satisfaction were included in the dimension of outcomes, having been in line with the findings of other studies (23).

In a study investigating the quality of rehabilitation center services based on the Donabedian model, the dimension of structure included facilities, resources, and systems, including beds and renovated wards, qualified specialists, including physiotherapists, occupational therapists, and specialists of professional training, education and experience, the bed ratio to the staff, and unique medical and technical equipment for patients. In addition, the care process at the rehabilitation center included diagnosis, treatment, prevention, and patient training activities, as well as social support and discharge programs. In terms of the dimension of outcomes, rehabilitation outcomes included improved patient knowledge, health status and behavior, as well as patient satisfaction that consisted of improved performance and the level of support expected by the patient after getting discharged (24).

In our study, continuous training was one of the major categories of the dimension of structure. Moreover, in line with our study, care was one of the categories of the dimension of process. In addition, professional excellence was one of the categories of the dimension of outcomes, which was resulted from scientific and professional maturity of the treatment staff in dealing with the unknown COVID-19 pandemic.

In the present study, crisis management was one of the categories needing further investigations and comprehensive planning. According to the participants, some subcategories of crisis management showed unfavorable status of crisis management, indicating haste and unpreparedness among managers in dealing with the crisis. In line with our findings, a study considered human resource management in the COVID-19 pandemic challenging and stated that the emergence of this situation requires managers to quickly enter the unknown world and do their best to help employees adapt to fundamental changes (25).

Running continuous educational programs in the mentioned hospital was considered one of the continuous and long-standing structures. Researchers in a study tried to investigate the effects of providing training courses in continuous intensive care in hospitals in 31 provinces of China. This meant to increase potentials for providing treatment and care services to patients in intensive care units (26).

Effective management and leadership in healthcare system were among the major process categories in our study, with effective leadership being more crucial right now than before the COVID-19 pandemic. In an article, it was stated that leadership must take steps to promote sustainable individual and team leadership skills as healthcare dramatically keeps changing during crises (27). In another study, the ability of the emergency department leadership in the COVID-19 pandemic was introduced as the key measure in clinical practice, which helps determine the modifiable structure and process leading to improved safety (13). Among the subcategories of the dimension of process, one can refer to uncertainties in treatment programs and changes in the treatment process of COVID-19 patients. A study similar to the present one considered unknown policies on treating COVID-19 patients the medical team's experience (7). Besides, providing care and treatment under unsafe conditions was one of the subcategories produced by data analysis. A study considered provision of care in the COVID-19 pandemic a horror story that happened despite the shortage of PPE, resulting in providing care services under unsafe conditions. Based on medical ethics, the need for protecting medical staff in providing treatment is an undeniable necessity (28, 29). One of the subcategories in the crisis management process was professional communication. A study considered improvements and use of communication infrastructures in establishing professional communication among medical staff as well as prevention of isolation of medical staff in the COVID-19 crisis very effective (30).

In this study, promoting nursing status was one of the subcategories of the dimension of outcomes. The participants unanimously stated that the scientific nature and practical competence of nurses in providing services during biological crises had been revealed to the public. This finding was contrary to an article indicating the inability of nursing leaders and nurses, compared to other medical staff, to show characteristics of nurses and importance of the nursing profession to the world in the COVID-19 pandemic, despite their competence (31). Another category was to quantitatively and qualitatively improve hospital services. In line with the present study, other studies reported changes in providing services at different hospitals. A study indicated that providing and expanding services to patients with mental health problems were among favorable outcomes in the management of the COVID-19 pandemic (32). In addition, another study reported that establishing virtual clinics was an outcome of promoting patient services (33).

Another challenging outcome of the components of the Donabedian model in this study was the satisfaction of COVID-19 patients and their families. In this category, individuals and families showed satisfaction or dissatisfaction with the services they received. A study in Ethiopia reported that COVID-19 patients' satisfaction with hospital services was very low (34). According to a study in Iran, COVID-19 patients asserted that the quality of services was based on the previous criteria for evaluating services as well as the features of agility, flexibility, and updating. In this study, the quality of services provided by hospitals of high rankings was lower than that of grade 2 and 3 hospitals (35).

In a study on the structure, process, and outcomes, using the Donabdin's model, the goals of the outcome was to assure the safety of the treatment staff, to reduce the number of clients, to lower hospitalization and mortality rates, and to prevent the spread of the disease (13). In our study, in addition to the acceptability of healthcare professionals, the most important outcomes were introduced to be professional development of the treatment staff as well as quantitative and qualitative development of the hospital and its services.

According to the research team, this study was the first one to have used the Donabedian model to investigate the dimensions and quality of medical services provided during the first wave of COVID-19 in Iran. Understanding and using components of this model in evaluating services provided can be considered a suitable reference for planning and eliminating shortcomings explained in each of the dimensions of this model. This will help hospitals get prepared to provide desired and standard services. Another strength of the present study was its use of the experiences and perspectives of physicians, managers, and patients in identifying factors determining the quality of care.

## Limitations

This study was conducted in a referral hospital in Tehran, so its results must not be generalized to other hospitals. In addition, experiences presented in this study were the results of the interviews conducted in the first wave of the COVID-19 pandemic to deal with the crisis. A study with multiple sites or of a longer duration could have led to different findings. Accordingly, new experiences may be gained in future COVID-19 waves and at the end of the pandemic, with the use of which improving the quality of hospital services. Thus, it is recommended that the causes of challenges be examined, and the strengths and weaknesses of the healthcare system be investigated in dealing with this pandemic. Based on the goals of our study, we used a limited number of patients and caregivers' families, so studies with higher sample sizes may offer different results. Due to the qualitative nature of this study, the difficulty in recruiting an adequate number of participants to reach data saturation was one of the limitations of this study. In addition, it is highly important to learn from the experiences of experts, such as senior managers, disaster health professionals, and healthcare policy makers, in this field in future studies.

## Conclusions

The present study was based on the Donabedian model, which qualitatively analyzed the conditions and factors making up the dimensions of the model in the services provided to patients in the first wave of the COVID-19 pandemic. Factors forming the dimension of structure included operational preparedness and continuous training. On the other side, effective management and leadership processes, as well as safe care and comprehensive care measures made up the dimension of process.

In addition, “professional excellence”, “quantitative and qualitative improvements in hospital services”, and “patient satisfaction” formed the dimension of outcomes. Furthermore, professional excellence as well as quantitative and qualitative improvements in hospital services and patient satisfaction formed the dimension of outcomes.

The results of this study indicate that to achieve more desirable outcomes, such as professional excellence, quantitative and qualitative improvements in hospital services, and acceptability of healthcare professionals (patient satisfaction and trust) in public health crises, more attention is needed to be paid to structures, such as operational (organizational) readiness, along with continuous training. In addition, improvements in organizational management processes (effective leadership), safe care, and comprehensive care measures should always be given special attention by managers. The results of this study can help managers understand more deeply how a public health crisis affects the structure of organizations providing care and treatment and how treatment processes must be followed in the organization. In addition, it can be used as a model for optimizing the structure and processes to improve the outcomes.

## Data availability statement

The raw data supporting the conclusions of this article will be made available by the authors, without undue reservation.

## Ethics statement

This study is part of the results of a large research project. The studies involving human participants were reviewed and approved by IR.BMSU.REC.1399.026. The patients/participants provided their written informed consent to participate in this study.

## Author contributions

MM and AP conceived, designed the work, and conducted the interviews. MM, AP, RK, and AE performed the analysis of interviews and feedback on the other interpretation in the expert panel. MM, AP, and RK wrote the first draft of the manuscript, critically reviewed for important intellectual content by AE and all of the authors. All authors reviewed the draft and approved the final version of the manuscript.

## Funding

This project was fully supported and funded by Baqiyatallah University of Medical Sciences, Tehran, Iran.

## Conflict of interest

The authors declare that the research was conducted in the absence of any commercial or financial relationships that could be construed as a potential conflict of interest.

## Publisher's note

All claims expressed in this article are solely those of the authors and do not necessarily represent those of their affiliated organizations, or those of the publisher, the editors and the reviewers. Any product that may be evaluated in this article, or claim that may be made by its manufacturer, is not guaranteed or endorsed by the publisher.
