# Supplementary material for: Factors determining the quality of health services provided to COVID-19 patients from the perspective of healthcare providers: Based on the Donabedian model
Source: Front Public Health. 2022 Nov 28;10:967431. doi: 10.3389/fpubh.2022.967431 (PMC9742228; doi:10.3389/fpubh.2022.967431)
Supplement: Supplementary file 1 [file Data_Sheet_1.DOC]

**Interview guide**

**1. Orientation**

The aim of our study was to explain the factors determining the quality of health services provided to COVID-19 patients. To do this, your consent to participate in the study is required, although you can interrupt the interview and withdraw from the study at any time. I have to record the interview. Then I transcribe them. Your name will not be mentioned in the report or article. First, fill out the informed consent form with demographic information including age, sex, and years of experience, educational status, and marital status.

**2. Primary questions**

Mention "your experience of caring for COVID-19 patients.

The next questions are based on the participants' experiences. Examples:

“Please describe a day you cared for a COVID-19 patient.”

**3. Main question**

“Please describe your experiences, with care; and descriptions of the critical care work system:

- Structures the questions focused on, for example (factors, human infrastructures, care and protection equipment and tools, as well as organization of human resources).
- Process for example (types of activities conducted by the employees, the way they conducted them based on instructions, the healthcare providers’ performance, and the way they were related to colleagues and patients in terms of providing care).
- Outcome for example (impact of the services and the quality of care on patients and the healthcare providers).

“What problems did/do you faced in the management of COVID-19?”

“What were the conditions of COVID-19 patients?”

**4. Probing question**

“What do you mean?”

“Would you please explain more?”

**5. Terminal phase**

The researcher's questions and discussions are over, please let me know if you have any other points or questions.

**Thank you for your participation in this study.**
