# Supplementary material for: Factors determining the quality of health services provided to COVID-19 patients from the perspective of healthcare providers: Based on the Donabedian model
Source: Front Public Health. 2022 Nov 28;10:967431. doi: 10.3389/fpubh.2022.967431 (PMC9742228; doi:10.3389/fpubh.2022.967431)
Supplement: Supplementary file 2 [file Table_1.DOCX]

Organizational readiness

Crisis management

Appropriate infrastructures

Benefiting from international experiences and assistance

**Continuous training**

Training and maneuvering programs

Trained personnel

**Effective management and leadership**

1. Human resource planning

2. Organization and coordination in the supply and distribution of human resources

3. Decision-making skills during crises

4. Professional communication under crisis conditions

5. The need for a united command

6. Employing voluntary forces

**Safe care**

1. Inadequate and unsuitable protection conditions

2. Inadequate environmental health conditions

**Comprehensive care measures**

1. Focusing on emotional needs

2. Care as a professional responsibility

**Quantitative and qualitative improvement in hospital services**

1. Structural development of the hospital

2. Promotion of specialized medical and educational services

3. Equipping the hospital with medical supplies

**Acceptability of Healthcare Professionals**

1. Patient trust

2. Patient satisfaction

**Professional excellence**

1. Competency promotion

2. Self-confidence

3. Professional promotion

**Outcome**

**Process**

**Structure**
